# Harnessing the placebo effect to enhance emotion regulation effectiveness and choice

**DOI:** 10.1038/s41598-023-29045-6

**Published:** 2023-02-09

**Authors:** Roni Shafir, Maya Israel, Luana Colloca

**Affiliations:** 1 Department of Pain and Translational Symptom Science, School of Nursing, University of Maryland, Baltimore, USA; 2 The School of Psychological Sciences, Tel Aviv University, Tel Aviv, Israel; 3 Department of Anesthesiology and Psychiatry, School of Medicine, University of Maryland, Baltimore, USA; 4 Center to Advance Chronic Pain Research, University of Maryland, Baltimore, USA

**Keywords:** Psychology, Health care

## Abstract

The placebo effect demonstrates how positive expectancies shape the effectiveness of various treatments. Across studies, placebo treatments are interventions (creams, pills, etc.) that are presented to individuals as, and are learned to be, beneficial for them. This study tested whether placebo-induced expectancies can be harnessed to improve individuals’ internal emotion regulation attempts. Participants implemented two types of distraction, an emotion regulation strategy involving attentional disengagement, to attenuate fear of pain. In a typical conditioning paradigm, the placebo-distraction was introduced as an effective strategy (verbal suggestion) and was surreptitiously paired with reduced pain (conditioning), whereas the control-distraction was introduced as noneffective and was surreptitiously paired with increased pain. As predicted, we found that during a later test phase, where pain intensity was identical, the placebo-distraction resulted in reduced self-reported fear of pain, relative to the control-distraction. Moreover, we utilized a robust behavioral choice measure, demonstrating increased preferences for the placebo-distraction. We additionally tested whether these effects generalize to a different emotional context of fear of unpleasant pictures. In that context, the placebo-distraction was as effective as the control-distraction, but was substantially preferred. This study demonstrates that the placebo effect can be expanded to include individuals’ internal attempts to influence their conditions.

## Introduction

When thinking about the power of the mind in shaping people’s experiences, the placebo effect often comes to mind. The placebo effect is perhaps one of the most outstanding manifestations of the link between individuals’ positive expectancies and actual improvement in outcomes. It demonstrates how treatments inherently inert become beneficial by forming positive expectancies regarding their impact^1,2^. Moreover, there is extensive placebo literature showing how forming positive expectancies can augment the effectiveness of already effective treatments^3–5^. In that sense, placebo effects are not limited to inert treatments, but also represent a powerful mechanism for boosting outcomes following the administration of active treatments^6,7^.

Over the last decades, a large portion of placebo research has been focused on pain, a complex experience that includes both physiological and psychological dimensions and is highly influenced by emotions and expectancies^8,9^. To maximize the effects of expectancies on pain experience, experimental placebo studies most commonly utilize conditioning paradigms, which combine verbal suggestion (i.e., explicitly suggesting to individuals that a certain treatment is effective) and conditioning (i.e., covertly pairing between the treatment and actual effective outcomes)^10–13^. For example, an inactive cream is presented as an analgesic and is administered to participants while, unknown to them, they receive reduced painful stimulations. By contrast, the control cream is presented as noneffective and is paired with increased painful stimulations. During a later test phase, although painful stimulations are identical in intensity, a reduction in self-reported, physiological, and neural indices of pain is still evident when the placebo (relative to the control) cream is administered due to participants’ positive expectancies for relief^12,14–16^. Strikingly, neural models of the placebo effect suggest that the administration of placebos in the context of pain modulation is associated with the release of neuropeptides (e.g., endogenous opioids) in the brain and the activation of brain regions involved in pain-processing and the formation of expectancies^6,17^.

Interestingly, a common feature that appears to be present across placebo studies is the focus on enhancing individuals’ positive expectancies exclusively regarding the effectiveness of *treatment interventions*, such as creams, pills, etc. In other words, the placebo itself is typically an intervention that is presented to individuals as, and is learned to be, beneficial for them. This raises two major theoretical considerations. First, the placebo treatment is a tool that is not always available to individuals (e.g., the placebo analgesic cream could be out of reach). Second, the use of the placebo treatment, rather than an internal action of individuals, is the trigger for individuals’ improvement in conditions. Could placebo-induced expectancies be harnessed to improve individuals’ internal attempts to improve their outcomes?

To answer this question, we turned to emotion regulation (ER), defined as individuals’ attempts to influence their emotional condition^18,19^. Specifically, we focused on distraction, a commonly used cognitive ER strategy^20–22^. Distraction implementation involves disengaging attention away from emotional information by producing unrelated neutral thoughts. Distraction has been shown effective in short-term modulation of emotions, even in high intensity emotional situations. This manifests in decreased negative experience^23,24^, reduced Late Positive Potential amplitudes (LPP, a neural marker of emotional processing)^25^, and stronger modulation of activation in brain areas associated with emotional responses, such as the amygdala^26,27^. Another notable advantage of distraction is that it requires minimal cognitive resources to operate^25,28^.

Hence, the first goal of the present study was to test for the first time whether placebo-induced expectancies could be built around an ER strategy, namely, distraction, instead of around a placebo intervention. To that end, we adopted the typical abovementioned conditioning paradigm. Participants were experiencing fear of possible painful shock^29–31^. Following a no-regulation baseline phase, where they were instructed to naturally experience their fear of possible painful shocks, they implemented two similar types of distractions^25,32^—thinking about writing letters versus thinking about drawing shapes, one served as the placebo-distraction and the other served as the control-distraction. The placebo-distraction was introduced as being effective in reducing fear and pain (verbal suggestion) and was surreptitiously paired with reduced painful stimulations (conditioning), whereas the control-distraction was introduced as noneffective and was surreptitiously paired with increased painful stimulations. During a later test phase, shocks were identical in intensity and participants’ fear of shocks in the two distraction conditions was measured. We predicted that (A) relative to a no-regulation baseline condition, the placebo-distraction would be more effective than the control-distraction in decreasing self-reported fear of shocks.

Importantly, beyond measuring self-reported fear under the two distraction conditions, we included a robust behavioral measure of individuals’ preferences between the two distractions. Specifically, we asked whether placebo-response expectancies would increase individuals’ behavioral choice for the placebo-distraction over the control distraction, when regulating fear of pain. Choice measures are prevalent in ER research seeking to understand individuals’ behavioral preferences for different ER strategies in varying emotional contexts^22,33–35^. Applying the typical regulatory choice task^36,37^, we asked participants to freely choose whether they wish to regulate their fear of painful shocks via the placebo-distraction or the control-distraction. We predicted (B) increased placebo-distraction over control-distraction choice when regulating fear of shocks.

The second goal of this study was to explore whether the effects of placebo-induced expectancies can be transferred from one domain to another. Specifically, we tested whether the increased effectiveness of, and preference for the placebo-distraction compared to the control-distraction, would generalize to a different emotional context of regulating fear of unpleasant pictures. Notably, as opposed to fear of shocks, which are aversive tactile stimulations, fear of unpleasant pictures involves a different domain of aversive visual stimulations.

There is evidence showing that placebo analgesics can be effective in contexts unrelated to pain, particularly when a conditioning (but not only verbal suggestion) manipulation was utilized to reinforce the expectancies for pain relief. For example, studies have found that placebo analgesics were also effective in attenuating negative emotions^38,39^. Consistent with these previous findings, we predicted that (C) relative to a no-regulation baseline condition, the placebo-distraction would be more effective than the control-distraction in decreasing self-reported fear of unpleasant pictures, and that (D) participants will show increased placebo-distraction over control-distraction preference when regulating fear of unpleasant pictures.

## Methods and materials

The study was conducted at Tel-Aviv University from March 2021 to November 2021, and was approved by the Institutional Review Board of Tel-Aviv University. All procedures were performed in accordance with the relevant guidelines and regulations. All participants signed an informed consent form before starting the study. Participants completed the study for course credits or monetary compensation (140 NIS).

### Participants

Thirty-five Israeli participants (mean age 24.43 years, 11 men) completed the study. Inclusion criteria included being 18–35 years old, speaking native Hebrew, and having normal vision. Exclusion criteria included being diagnosed with ADHD/anxiety/mental/neurological/cardiological disorder, and taking psychiatric medications. Two participants were excluded from data analysis because they disclosed the placebo induction manipulation; during debriefing, they explicitly told the experimenter that shocks intensity differed between the two conditioning blocks. One additional participant was excluded because it turned out she was not a Hebrew native speaker (i.e., one of the a-priori inclusion criteria for participating). Thus, the final sample consisted of thirty-two Israeli, native Hebrew-speaking participants (mean age 24.31 years, 10 men). Most participants (29 out of 32) were students at Tel-Aviv University, among them 17 psychology students.

### Stimuli

We used both electrical shocks and pictures as stimuli. Electrical shocks were administered using a STIMSOLA device (BIOPAC Systems Inc.) delivering shocks through two electrodes placed on the participants’ lower left arm. For the generalization part, we used thirty-five unpleasant pictures from several previously validated pictorial datasets (with comparable normative valence and arousal ratings): International Affective Picture System^40^, GAPED^41^, EmoPicS^42^, and Nencki Affective Picture System^43^. We selected unpleasant pictures that were moderately high in intensity (mean arousal of 6.48, mean valence of 2.24) in order to match the moderate intensity of the shocks that were delivered during the baseline no-regulation (experience) phase, the test phase and the choice phase (see below). The pictures depicted a variety of unpleasant contents, such as sadness, disgust, and fear (see Supplementary Table 1 for a full list of the pictures).

### Procedure

**Figure 1:**
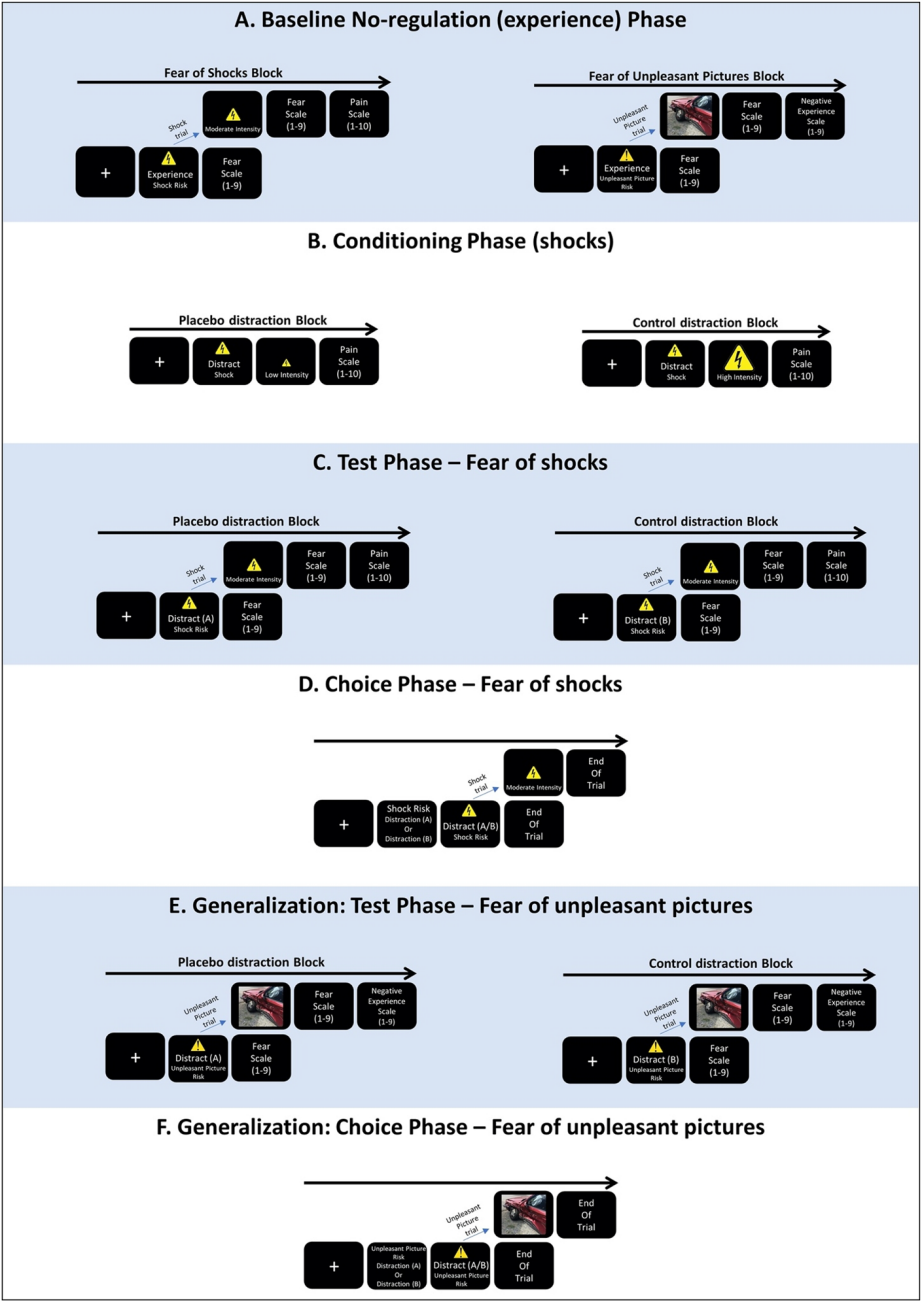
Study overview: schematic trial structures of the different study phases. (**A**) The baseline no-regulation (experience) phase included two blocks—a fear of shocks block and a fear of unpleasant pictures block. Participants were asked to allow natural thoughts and feelings to arise while anticipating a possible shock/unpleasant picture. In trials with an electric shock/an unpleasant picture, they were asked to continue allowing their feelings to naturally arise. (**B**) The conditioning phase included two counterbalanced blocks—one for the placebo-distraction, which was presented as effective and paired with low intensity shocks, and one for the control-distraction, which was presented as not effective and paired with high intensity shocks. (**C**) The test phase included two counterbalanced blocks—one for the placebo-distraction and one for the control-distraction. These blocks were identical to the fear of shocks block in phase A, except that here participants implemented the two distractions instead of the experience instruction. (**D**) This phase included one block where participants freely chose between the two distractions while anticipating a possible shock. (**E**) The generalization test phase included two counterbalanced blocks—one for the placebo-distraction and one for the control-distraction. These blocks were identical to the fear of unpleasant pictures block in phase A, except that here participants implemented the two distractions instead of the experience instruction. (**F**) The generalization choice phase was identical to phase D, except that here participants freely chose between the two distractions while anticipating a possible unpleasant picture. In compliance with copyright laws, the picture used for this illustration is similar to, but not taken from the set of pictures presented in the study.

Once participants were deemed eligible, the study began. The study included several phases, as elaborated below (see also Fig. 1. For a study overview). First, ten electroencephalogram (EEG) electrodes were attached to participants’ scalps. The electrodes were in fact not recording brain activity. Rather, they served as part of a cover story that the study was focused on exploring the neural mechanisms underlying regulatory strategies that have been shown effective versus ineffective in reducing pain and fear.

All experimental instructions were provided verbally. However, to maximize adherence to instructions and consistency across participants, the trained experimenter was reading the instructions to participants, word by word.

#### Calibration

First, participants were asked to report three levels of their individual pain intensity—low, moderate, and high. They were falsely told that the calibration was aimed at tailoring an individual shock intensity level, which will remain fixed during the study. The intensity of the electric shocks was gradually increased by the experimenter until participants reported a low intensity level, namely, slightly unpleasant, requires little effort to tolerate, and equals the number 2 on a 1 (= minimally painful) to 10 (= unbearable) pain intensity scale. Then, the intensity was further increased until participants reached a moderate intensity level, namely, more unpleasant and requiring more effort to tolerate, relative to the low intensity, and equals the number 5 on the pain intensity scale. Last, the intensity was further increased until participants reached a high intensity level, namely, intense and requiring more effort to tolerate, relative to the moderate intensity, though still at a tolerable level, and equals the number 8 on the pain intensity scale. Importantly, the low and the high intensity levels were later used in the conditioning phase, for the placebo-distraction and the control-distraction blocks, respectively. The moderate intensity was used for the baseline no-regulation (experience) phase, the test phase, and the choice phase.

##### Baseline no-regulation (experience) phase

Following calibration, participants performed two 30-trials baseline no-regulation (experience) blocks: a fear of shocks block followed by a fear of unpleasant pictures block.

For the fear of shocks block, participants were told that in each trial, they will be at risk of receiving an electric shock. While anticipating a possible shock, they were asked to implement an “experience” instruction, namely, to allow natural thoughts and feelings (such as fear, tension, etc.) to arise, without blocking or changing what they feel. In trials where they receive an electric shock, they were asked to continue implementing the experience instruction, namely, to allow the unpleasant feeling of pain to naturally arise (see Supplementary Information 1 for detailed instructions). The experimenter made sure that participants understood the experience instruction properly before the task started, by asking them to talk out loud how they implemented it. Following 2 practice trials with sounds simulating shocks, the task started.

Each of the 30 trials began with a fixation cross (2100–2900 ms), followed by an *implementation screen* (3000 ms) presenting the words “risk of an electrical shock”, together with an electric shock danger warning sign and the instruction to be used—the word “experience”. Participants were asked to start implementing “experience” when this screen was presented, and were told that an electric shock could occur at any time while the implementation screen is presented. In trials where there was no shock, a black screen was then presented (400/600/800 ms), followed by a fear scale (until response) in which participants rated how much fear they felt while anticipating a possible shock and implementing “experience” in that trial (1 = I experienced no fear and 9 = I was very afraid). In trials where an electric shock occurred (10 in total), the implementation screen varied in duration (400/900/1500/2100 ms) and was followed by the delivery of an electric shock that also varied in duration (500/100/1500/2000 ms). Then, a black screen was presented (400/600/800 ms), followed by the 1–9 fear scale (until response), as well as the 1–10 pain scale (until response) used during the calibration.

To maximize fear, participants were not informed about the chances of receiving a shock (i.e., in one-third of the trials) and were told that shocks could appear at any time after the implementation screen presentation and could be of different durations. To increase the salience of the fear of shocks, we additionally included 5 “safe trials” (distributed pseudo-randomly in the block), where participants knew they had no risk of receiving a shock. These trials began with a fixation cross (2500 ms) on a blue-colored background, signaling that the trial was a safe trial, followed by the presentation of the words “safe trial”.

The fear of unpleasant pictures block included 30-trials and was identical to the previous experience—fear of shocks block in all aspects, except for two changes. First, instead of anticipating a possible shock, here participants were anticipating a possible unpleasant picture. Again, they were asked to allow natural thoughts and feelings to arise while anticipating a possible unpleasant picture, without blocking or changing what they feel. In case of an unpleasant picture presentation, participants were asked to continue allowing the unpleasant feelings in response to the picture to naturally arise (see Supplementary Information 1 for detailed instructions). Second, in trials where an unpleasant picture appeared, the pain scale was replaced with a negative experience scale (until response) where participants rated their negative experience in response to the picture, ranging from 1 (= not negative at all) to 9 (= very negative).

##### Conditioning phase (shocks)

This phase included two counterbalanced 10-trials blocks, one for each distraction type (thinking about writing letters versus thinking about drawing shapes).

Before each block, participants were taught how to implement the specific distraction type and received fictive information regarding its efficacy. Specifically, the placebo-distraction (whether letters or shapes, counterbalances across participants) was described as a strategy that was found very effective in reducing fear and pain. The experimenter further provided participants with a bogus scientific description of the underlying neural mechanisms that explain why the strategy is effective. In addition, the experimenter explained that studies show that even when people feel that they are not very successful at implementing this distraction, still it is highly effective. By contrast, for the control-distraction, the experimenter told participants that it was found not effective in reducing fear and pain, and provided them with a bogus scientific description of the underlying neural mechanisms that explain why it was ineffective. All explanations were slightly modified according to which distraction type served as the placebo-distraction versus the control-distraction (see Supplementary Information 1 for detailed explanations).

Following each distraction type description, participants had 2 practice trials with sounds simulating shocks. Participants were told that they will perform a training block for each distraction type in order to implement the strategy better, and that in this block they will receive an electric shock in every trial. Unknown to participants, we used the low and high intensity level shocks for the placebo-distraction and the control-distraction blocks, respectively.

Each trial in the two conditioning blocks began with a fixation cross (2100–2900 ms), followed by an *implementation screen* (3000 ms) presenting the words “electrical shock”, together with an electric shock danger warning sign and the distraction type to be used in that block—the words “letters distraction” or “shapes distraction”. Participants were asked to start implementing the strategy when this screen was presented, until the end of the trial. Then, a black screen was presented (50–150 ms), followed by the delivery of an electric shock (2000 ms). After another black screen (400/600/800 ms), participants rated their pain on the 1–10 pain scale (until response).

##### Test phase—fear of shocks

This phase included two counterbalanced 30-trials blocks, one for each distraction type. These two blocks were identical to the experience—fear of shocks block in all aspects, except that instead of implementing the experience instruction, here participants implemented the placebo-distraction and the control-distraction while anticipating a possible shock.

The experience—fear of shocks block, the placebo-distraction test block, and the control-distraction test block, each included a total of 10 trials with shocks. Shock trials were distributed pseudo-randomly, with the only rule being that the first/second trial must be a shock trial. This was done because we wished participants to receive the same moderate shock intensity level for both distraction types early at the beginning of the test blocks. This way, differences between the strategies could be attributed to the placebo effect, rather than to a continuation of the previous conditioning phase, where shock intensities differed.

##### Choice phase—fear of shocks

This phase included a 15-trials block. Following 2 practice trials, the task started. Each trial began with a fixation cross (2100–2900 ms), followed by a *choice screen* (until response) presenting the words “risk of an electrical shock”, together with the two distraction types participants could choose—“letters distraction” or “shapes distraction”. Participants were asked to freely choose the strategy they think would be most effective for them in regulating their fear (see Supplementary Information 1 for detailed instructions), using two keys ‘a’ or ‘l’ (assignment of keys to the two distraction types was counterbalanced across participants). Following regulatory choices, the implementation screen was presented (3000 ms in trials with no shocks, 400/900/1500/2100 ms in trials with shocks) and participants were asked to start implementing their chosen distraction type while anticipating a possible shock. In trials with shocks (5 in total), the implementation screen was followed by the delivery of an electric shock (500/100/1500/2000 ms). Both the implementation screen in no shock trials and the shock delivery in shock trials, were followed by a black screen (400/600/800 ms), followed by a screen presenting the words “end of trial” (1500 ms).

Again, to maximize fear, participants were not informed about the chances of receiving a shock and were told that shocks could appear at any time after the implementation screen presentation and could be of different durations. Three “safe trials” (distributed pseudo-randomly in the block) were also included.

##### Generalization: test phase—fear of unpleasant pictures

After completing the choice phase—fear of shocks, we tested whether the placebo effect generalizes to the context of fear of unpleasant pictures. This phase included two counterbalanced 30-trials blocks, one for each distraction type. These two blocks were identical to the experience—fear of unpleasant pictures block in all aspects, except that instead of implementing the experience instruction, here participants implemented the placebo-distraction and the control-distraction while anticipating a possible unpleasant picture.

Similar to the fear of shocks blocks, the exprerience—fear of unpleasant pictures block, the placebo-distraction generalization test block, and the control-distraction generalization test block, each included a total of 10 trials with unpleasant pictures. Picture trials were distributed pseudo-randomly, with the only rule being that the first/second trial must be an unpleasant picture trial. We created three 10-pictures lists of pictures with nearly identical average valence and arousal ratings (see Supplementary Table 1 for details). For each participant, the lists were randomly assigned to the three blocks (experience, placebo-distraction, control-distraction). Thus, across participants, each picture had an equal chance of being selected for each of the three conditions.

##### Generalization: choice phase—fear of unpleasant pictures

This phase included a 15-trials block which was identical to the choice phase—fear of shocks block in all aspects, except that here participants chose which strategy they wish to implement while anticipating a possible unpleasant picture (see Supplementary Information 1 for detailed instructions, Supplementary Table 1 for pictures list).

To ensure that participants adhered to the experimental instructions, at the end of the study they were asked to write down and describe in their own words what they thought about when implementing each of the instructions, namely, experience—fear of shocks, experience—fear of unpleasant pictures, and the two distraction types. Based on these written descriptions, adherence to both distraction types was 100%, mitigating the concern that participants did not implement the control-distraction because it was presented as, and learned to be, ineffective. For the experience instructions, levels of adherence were very high (93.75%).

### Statistical analyses

The sample size was *pre-determined* in a power analysis using MorePower 6.0^44^. Specifically, although the effect sizes observed in similar previous studies comparing the effectiveness of different ER strategies (e.g., η_*p*_^2^ = 0.63^32^) or the preference for different ER strategies (e.g., η_*p*_^2^ = 0.75^37^) were typically very large, we chose a conservative approach of using a smaller estimated effect size of η_*p*_^2^ = 0.14. The estimated effect size, η_*p*_^2^ = 0.14, was calculated for the repeated measures analysis of variance (ANOVA) with Instruction-Type (experience, placebo-distraction, control-distraction) as a three-level repeated-measures factor. Applying the conventional high power of 0.8 and an alpha of 0.05, the analysis indicated that a sample of 32 participants was required to detect a reliable effect. Considering a 10% exclusion rate, we decided to run a total of 35 participants.

The effectiveness of the two distractions in both fear contexts was examined using a one-way repeated measures ANOVA with Instruction-Type (experience, placebo-distraction, control-distraction) as a repeated-measures factor. These analyses included only trials with no shocks/unpleasant pictures, because the presence of shocks/unpleasant pictures could influence fear reports occurring at the end of the trial. In cases where the sphericity assumption was violated, as indicated by Mauchly’s Sphericity Test, we reported the Greenhouse–Geisser adjusted p-values^45^. We implemented planned contrasts between each distraction and the experience condition, and directly between the two distractions. The preference for the placebo-distraction over the control-distraction in both fear contexts was examined using a one-sample *t* test comparing participants’ average percentage of placebo-distraction choice to a 50% no preference rate. A significance level of *p* < 0.05 was selected. All analyses were conducted using JASP (0.14.1.0).

## Results

### Calibration

To confirm that the three shock intensity levels (low, moderate, high) participants reported during the calibration differed, we performed a one-way repeated measures ANOVA with Shock-Level in voltage (low, moderate, high) as a repeated-measures factor. As expected, we found a significant effect [*F*(2,62) = 489.36, Greenhouse–Geisser *p* < 0.001, η_p_^2^ = 0.94], where the low intensity level (M = 19.22, SE = 0.95, 95% Confidence Interval (CI) [17.17, 21.26]) was lower than the moderate intensity level (M = 23.69, SE = 1.03, 95% CI [21.64, 25.73]) [*t*(62) = 16.93, *p* < 0.001], and the moderate intensity level was lower than the high intensity level (M = 27.47, SE = 1.03, 95% CI [25.42, 29.51]) [*t*(62) = 14.32, *p* < 0.001].

### Manipulation check—pain during the conditioning phase

First, we confirmed that the conditioning manipulation was effective, namely, that participants felt increased pain levels during the conditioning phase in the control-distraction condition (which was associated with high intensity shocks), relative to the placebo-distraction condition (which was associated with low intensity shocks). To that end, we employed a one-way ANOVA with Distraction-Type (placebo, control) as a repeated-measures factor. This analysis revealed the expected significant Distraction-Type effect [*F*(1,31) = 106.87, *p* < 0.001, η_p_^2^ = 0.78], with higher pain ratings for the control-distraction (M = 6.82, SE = 0.30, CI [6.19, 7.45]), relative to the placebo-distraction (M = 3.65, SE = 0.32, 95% CI [3.02, 4.28]). This trend was evident in 96.88% (31/32) of the participants.

#### Is the placebo-distraction more effective in reducing fear of shocks?

**Figure 2:**
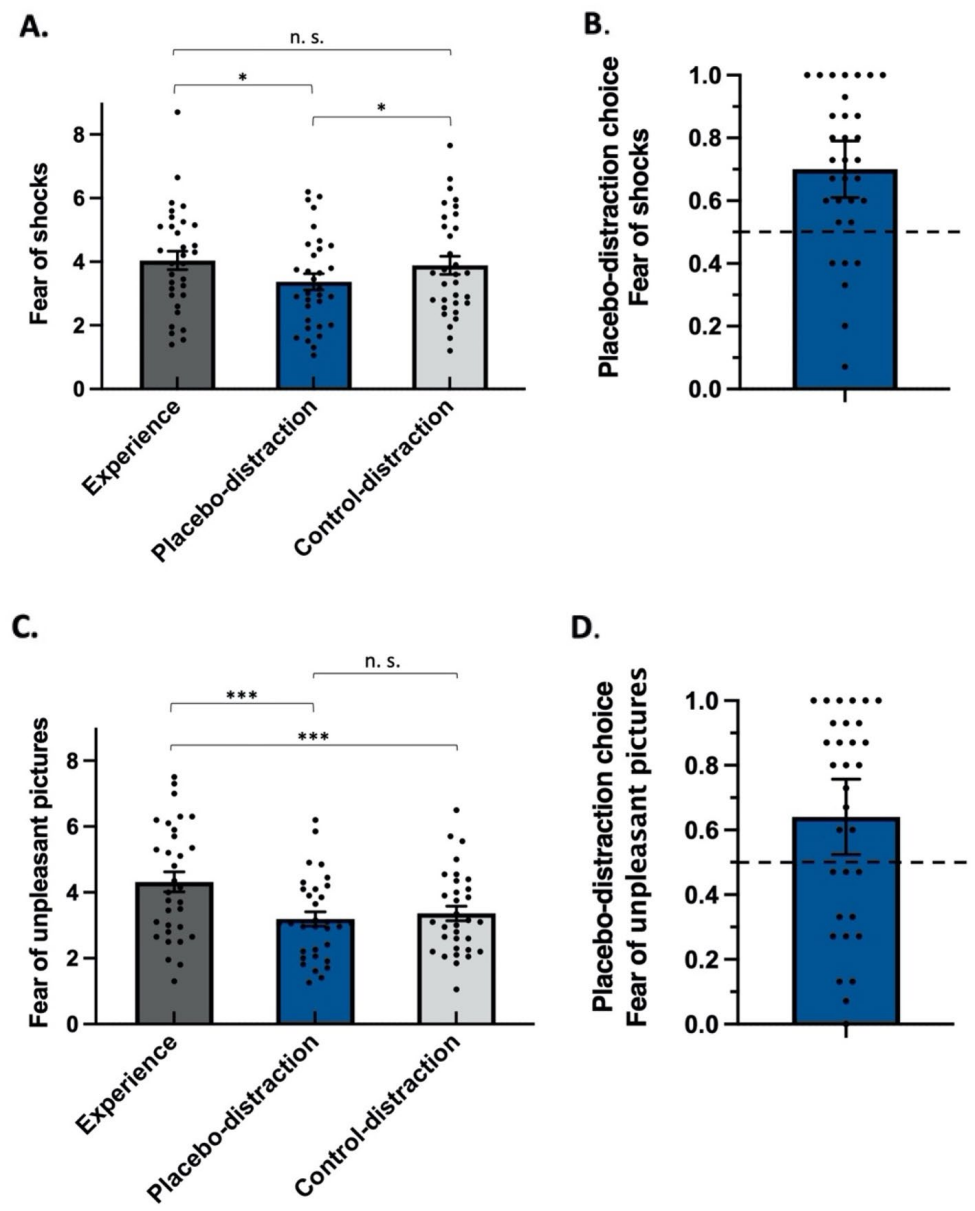
Study results. Self-reported fear of shocks (y-axis) (**A**) and fear of unpleasant pictures (y-axis) (**C**) for experience, placebo-distraction, and control-distraction. Higher values denote enhanced fear of shocks/unpleasant pictures. *p < 0.05; **p < 0.01; ***p < 0.001. Error bars represent standard errors. (**B**) Percentage of trials (y-axis) participants behaviorally chose the placebo-distraction over the control-distraction when regulating fear of shocks and (**D**) when regulating fear of unpleasant pictures. The bars represent 95% CIs. Note that most individual points fall above the horizontal lines, which represent a 50% "no preference" rate.

Consistent with our prediction that the placebo-distraction would be more effective in reducing participants’ fear of possible shocks, relative to the control-distraction, we found a significant Instruction-Type effect [*F*(2,62) = 3.80, Greenhouse–Geisser *p* = 0.046, η_p_^2^ = 0.11, see Fig. 2A], where relative to the no-regulation experience condition (M = 4.04, SE = 0.29, 95% CI [3.49, 4.60]), the placebo-distraction (M = 3.37, SE = 0.26, 95% CI [2.81, 3.93]) effectively reduced fear of possible shocks [*t*(62) = 2.63, *p* = 0.01], whereas the control-distraction (M = 3.89, SE = 0.29, 95% CI [3.34, 4.47]) did not [*t*(62) < 1, *n.s.*]. A direct comparison between the two distractions revealed that the placebo-distraction was more effective than the control-distraction [*t*(62) = 2.04, *p* = 0.046].

While the central target of the strategies in this study was the regulation of fear of possible shocks, we also conducted a secondary analysis exploring the impact of the manipulation on pain regulation. Specifically, we tested whether in trials that included shocks during the test phase, the placebo-distraction resulted in decreased pain ratings, relative to the control-distraction. The effect of Instruction-Type (experience, placebo-distraction, control-distraction) was non-significant [*F*(2,62) = 2.08, *n.s.*]. Nonetheless, the trend in means was in the expected direction, with lower pain ratings in the placebo-distraction (M = 4.96, SE = 0.33, 95% CI [4.34, 5.57]), relative to the control-distraction (M = 5.44, SE = 0.34, 95% CI [4.82, 6.06]) and the no-regulation experience (M = 5.17, SE = 0.24, 95% CI [4.56, 5.79]) conditions. Note that this analysis should be treated with caution. Because only one third of the trials included actual shocks, this analysis is based on only 10 trials per condition.

#### Is the placebo-distraction behaviorally preferred when regulating fear of shocks?

Providing behavioral evidence for the effectiveness of the placebo-distraction, compared to the control-distraction, one sample *t* test showed that participants’ preference for the placebo-distraction differed from a 50% no preference rate [*t*(31) = 4.50, *p* < 0.001, see Fig. 2B]. 81.25% (26/32) of the participants showed a preference for the placebo-distraction over the control-distraction, with an average placebo-distraction choice of 70.00% (SE = 4.4%, 95% CI [60.9%, 79.1%]).

### Generalization findings

#### Is the placebo-distraction more effective in reducing fear of unpleasant pictures?

Next, we tested whether the placebo-distraction was more effective in reducing participants’ fear of unpleasant pictures, relative to the control-distraction. We found a significant Instruction-Type effect [*F*(2,62) = 18.09, Greenhouse–Geisser *p* < 0.001, η_p_^2^ = 0.37, see Fig. 2C]. However, in this case, relative to the no-regulation experience condition (M = 4.32, SE = 0.30, 95% CI [3.81, 4.83]) both the placebo-distraction (M = 3.19, SE = 0.22, 95% CI [2.68, 3.70]) [*t*(62) = 5.58, *p* < 0.001] and the control-distraction (M = 3.36, SE = 0.22, 95% CI [2.85, 3.87]) [*t*(62) = 4.73, *p* < 0.001] effectively reduced fear of unpleasant pictures. The placebo-distraction did not differ from the control-distraction [*t*(62) < 1, *n.s.*].

Similar to the secondary analysis of pain regulation, we conducted a secondary analysis exploring whether in trials that included unpleasant pictures during the test phase, the placebo-distraction resulted in decreased negative experience ratings, relative to the control-distraction. Here, we found a significant effect [*F*(2,62) = 7.49, *p* = 0.001, η_p_^2^ = 0.20], where relative to the no-regulation experience condition (M = 6.29, SE = 0.20, 95% CI [5.84, 6.74]), both the placebo-distraction (M = 5.50, SE = 0.25, 95% CI [5.05, 5.95]) [*t*(62) = 3.87, *p* < 0.001] and the control-distraction (M = 5.87, SE = 0.22, 95% CI [5.42, 6.32]) [*t*(62) = 2.05, *p* = 0.045] effectively reduced negative experience ratings. The placebo-distraction was marginally more effective than the control-distraction [*t*(62) = 1.82, *p* = 0.074]. Note that this analysis should be treated with caution due to only 10 trials per condition.

#### Is the placebo-distraction behaviorally preferred when regulating fear of unpleasant pictures?

Similar to the behavioral choice results we observed with fear of shocks, while anticipating possible unpleasant pictures, participants’ preference for the placebo-distraction differed from a 50% no preference rate [*t*(31) = 2.44, *p* = 0.02, see Fig. 2D]. 62.5% (20/32) of the participants showed a preference for the placebo-distraction over the control-distraction, with an average placebo-distraction choice of 64.00% (SE = 5.7%, 95% CI [52.3%, 75.7%]).

Out of all the above reported results, we found one case of an interaction with the type of distraction used for the placebo condition. Specifically, participants chose the placebo-distraction more when it involved thinking about writing letters, relative to thinking about drawing shapes [*t*(30) = 2.13, *p* = 0.042].

The current sample was unbalanced in terms of sex, with 10 men and 22 women. Nonetheless, following recent recommendations to account for sex effects in placebo research^46^, we additionally report that the sex of the participants (women versus men) did not interact with any of the results (all *p*’s > 0.15).

## Discussion

Placebo research typically focuses on treatment interventions, which are described to individuals as beneficial for them and are also learned to be effective via conditioning. This study demonstrated for the first time that a placebo treatment could be replaced with an ER strategy that individuals actively implement, namely, distraction, so as to improve its effectiveness. The placebo-distraction was presented to participants as being effective in reducing fear and pain, and was surreptitiously paired with reduced painful stimulations, whereas the control-distraction was introduced as noneffective and was surreptitiously paired with increased painful stimulations. Consistent with our prediction, during a later test phase, where shocks were identical in intensity, the placebo-distraction was more effective than the control-distraction in reducing self-reported fear of pain. Moreover, when choosing between the two distractions, participants substantially preferred the placebo-distraction over the control-distraction for regulating their fear of pain.

Relatively little attention has been dedicated to exploring the relations between placebos and cognitive ER strategies, with no study replacing the placebo treatment with a cognitive ER strategy. When categorizing the existing literature, we identified two types of relevant studies. The first includes studies that compared between placebos and cognitive ER strategies, in terms of their effectiveness and required cognitive resources. For instance, one study^47^ looked at neural networks involved in placebo versus reappraisal^22^ implementation in response to repulsive pictures. In three different sessions, participants passively viewed the pictures, implemented reappraisal by telling themselves that the pictures are unreal, and received a placebo pill that was introduced as a disgust-reducing drug. While both placebo and reappraisal reduced self-reported disgust, relative to passive viewing, patterns of neural activation differed. Specifically, reappraisal implementation was associated with increased activation in brain regions linked to cognitive control, whereas the placebo decreased activation in these regions. Another study compared a reappraisal group, where participants were interpreting unpleasant pictures in a less negative manner, and a placebo group, where participants passively viewed unpleasant pictures while an electrode (in fact sham) that is suggested to reduce emotional arousal was activated^48^. Results showed that both placebo and reappraisal reduced self-reported unpleasantness, as well as activity in brain regions associated with emotional processing. This is congruent with theoretical accounts that consider meaning-making—an essential part of reappraisal, to be a significant component of the placebo effect^7^. Nonetheless, again, only reappraisal required the activation of neural cognitive resources.

Focusing on distraction, in another study^49^ participants underwent two sessions where they performed a cognitive distractive task while receiving painful heat stimuli. In the placebo session, they performed the task with a placebo “analgesic” cream, whereas in the control session, they performed the task with a nonanalgesic cream. Participants reported reduced pain in both sessions, but the reductions were additive, suggesting that placebo and distraction might have independent analgesic effects. The authors concluded that as opposed to distraction, placebo analgesia does not require executive resources to operate. Together, these prior studies suggest that when directly compared with cognitive ER strategies, placebo treatments seem to require less cognitive effort to change emotional reactions.

The second type of studies exploring the intersection between placebos and ER strategies lies within the ER literature. Viewed from an ER perspective, one important factor required to successfully regulate emotions is ER self-efficacy, that is, beliefs about one’s ability to control emotions^50,51^. To our knowledge, two studies have used placebo drugs to enhance participants’ beliefs about their ability to successfully regulate emotions^52,53^. Specifically, in both studies participants were receiving a drug before being exposed to unpleasant contents. Participants in the placebo group were told that a common side effect of the drug involves enhanced emotional control, whereas participants in the control group were informed of no additional side effects. Results in both studies showed reduced self-reported negative mood in the placebo, compared to the control groups. While providing important insights, in these studies positive expectancies regarding ER success were again built around a treatment (in this case a drug) that is suggested to boost ER ability. Thus, here we provide the first evidence for how the placebo effect can be utilized to enhance ER success, rather than a treatment that in turn enhances ER success.

The second goal of this study was to explore whether the increased effectiveness of, and preference for, the placebo-distraction compared to the control-distraction, would generalize to a different domain of regulating fear of unpleasant pictures. We found partial support for a generalization effect. Specifically, the placebo-distraction was as effective as the control-distraction when regulating fear of unpleasant pictures, but was behaviorally preferred over the control-distraction in that context. Given that the effect sizes of behavioral ER choice measures are generally more robust than those of self-reported effectiveness measures^22^, as also observed in the current study, it could be that the study was not powered enough to detect a generalization effect for the self-reported fear of unpleasant pictures. Future studies are needed to further explore the extent to which the effects we observed generalize. More broadly, it would be important to replicate the current results with larger sample sizes in future studies.

The current findings have important implications. From a placebo research perspective, our results emphasize that the investigation of placebo effects does not have to be limited to treatment interventions, but can rather be expanded to include individuals’ internal attempts to influence their conditions. Clearly, this may be more challenging for researchers and for clinicians who treat patients. Compared to leading individuals to attribute an improvement in conditions to an intervention (e.g., a placebo analgesic cream that reduces pain), leading them to attribute an improvement in conditions to an internal action (e.g., distracting thoughts from the pain) can be tricky. This is mainly because when it comes to internal actions, individuals have a sense of how successful they are in implementing them. For instance, one can feel a failure to distract thoughts from pain during conditioning, which makes it hard to reliably attribute the reduction in pain to distraction attempts. This was a central reason for choosing to use distraction. Specifically, distraction is known to be a particularly effective ER strategy in the short-term^24,25,54^, which reduces the risk of participants’ uncovering the conditioning manipulation. If we were to choose a strategy that is less effective, participants would have been more suspicious during trials in the conditioning phase where the strategy was less successful but still resulted in reduced pain.

A possible way to overcome this challenge could be using Open Label Placebos (OLPs), i.e., placebos that are provided without deception by explaining to participants about the placebo effect and its positive influence on outcomes^55^. Beyond the importance of minimizing ethical concerns associated with deceptions, OLPs given as an adjuvant treatment would allow individuals to be informed about the influence of positive expectancies on the effectiveness of their internal actions, such as ER implementation, putting aside individuals’ concerns about how successful they are in implementing these actions. This would also open a window for testing the influence of placebo-induced expectancies on other, less effective in the short-term strategies, such as reappraisal^22^.

From an ER research viewpoint, potent manipulations adopted from placebo research, such as conditioning, represent a novel approach for improving the effectiveness of ER strategies, as well as individuals’ drive to choose to implement these strategies^56^. This is of high importance given that ER implementation can fail in various situations, for instance, under fatigue or acute stress^57–59^, and some individuals, e.g., those who suffer from major depression, fail to benefit from generally effective ER strategies^60^. Although as discussed above, two studies have shown that regulation success can be boosted when individuals believe a drug could enhance their emotional control^52,53^, no study took a step forward by reinforcing ER implementation with conditioning. Because conditioning involves a direct experience of better learned outcomes, it has been shown to be a particularly powerful means of inducing reinforced expectancies and positive outcomes^61^. Future ER studies seeking to boost ER success could therefore utilize both verbal suggestion and conditioning procedures. To further strengthen the impact of placebo-induced expectancies on ER effectiveness, future studies could use longer conditioning blocks that were shown to produce more robust placebo effects^62^, enhance the perceived competence, expertise, and reassurance of the experimenter^63^, and so forth.

Several limitations of the present study should be noted. First, self-reported measures are naturally prone to reporting biases. To minimize demand characteristics, we used a cover story that the study was exploring the neural mechanisms underlying different ER strategies, while seemingly recording EEG. This way, participants were led to believe that their subjective experience was not our focus of interest. More importantly, in addition to self-reported fear of shocks and fear of unpleasant pictures, we adopted a behavioral measure from ER research that concerns preferences for the placebo-distraction in both domains. Nonetheless, future studies should utilize objective measures (e.g., neural) as well, to further minimize the influence of demand characteristics.

Second, while the present findings are novel in the sense that they show for the first time how cognitive ER can benefit from placebo-induced expectancies, the question remains what made the placebo-distraction more effective, as well as preferred. One possible explanation is that participants put minimal or no effort into implementing the control-distraction, because it was presented as, and learned to be, ineffective. This seems unlikely for two main reasons. First, while this explanation is consistent with the fear of pain results, where the control-distraction did not differ from the no-regulation experience condition, it is inconsistent with the finding that the control-distraction effectively reduced fear of unpleasant pictures, relative to the no-regulation experience condition. Second, as mentioned above, participants’ adherence to both distraction types reached 100%.

Nonetheless, it could be that participants were more motivated, and thus put considerably more effort into implementing the placebo-distraction. To test that, beyond measuring participants’ self-reported motivation and effort, future studies can utilize behavioral, physiological, and neural measures. For example, studies could use online neural indices of cognitive effort, such as the frontal-LPP, an event related potential component that was shown to indicate the degree of cognitive effort associated with ER implementation^25,64^. Finding increased frontal-LPP amplitudes during the placebo-distraction implementation, for example, would suggest enhanced cognitive effort. Future studies should explore this and other potential underlying explanations.

Third, given that the study was aimed to address the question of whether placebo-induced expectancies can boost distraction in the face of unpleasant emotions (namely, fear), we did not include neutral stimuli. Rather, the no-regulation experience condition, where participants experienced unpleasant emotions without regulating, served as a baseline. In future studies, an additional important baseline measuring responses to neutral stimuli, could be added to the standard, well-established no-regulation condition.

Fourth, we found that participants chose the placebo-distraction more when it involved thinking about writing letters, relative to thinking about drawing shapes. It is important to note that this was the only case where the type of distraction used in the placebo condition interacted with the findings. Nonetheless, future studies could explore potential characteristics of ER strategies that make them more susceptible to placebo manipulations—similar to how classic placebo studies explore how specific characteristics of the placebo treatment (e.g., its color) enhance the placebo effect.

Last, while we measured participants’ preferences for the placebo-distraction over the control-distraction, we did not measure the experiential consequences of s’ choices between the strategies, namely, their fear ratings after implementing their chosen distraction. It has been demonstrated that when given the opportunity to choose between two (inert) treatments, as opposed to no choice opportunity, participants show increased placebo effects^65–67^. Thus, it would be interesting for future studies to test whether the opportunity to choose between ER strategies enhances the effectiveness of the placebo strategy.

In conclusion, the present study provides a unique empirical examination of whether placebo-induced expectancies can be harnessed to improve individuals’ internal attempts to cognitively regulate their emotions. This study employs, in addition to self-reported ER effectiveness, a robust behavioral measure that is relatively new to placebo research, namely, individuals’ preferences for the placebo ER strategy. Broadly, this work opens an avenue for bridging placebo research with ER research, and further expanding the scope of what can serve as a placebo treatment.

## Supplementary Information

Supplementary Information 1.

Supplementary Information 2.

## Data Availability

All data analyzed for the study is included in the Supplementary Information.
