# Supplementary material for: Harnessing the placebo effect to enhance emotion regulation effectiveness and choice
Source: Sci Rep. 2023 Feb 9;13:2373. doi: 10.1038/s41598-023-29045-6 (PMC9911767; doi:10.1038/s41598-023-29045-6)
Supplement: Supplementary file 1 — Supplementary Information 1. [file 41598_2023_29045_MOESM1_ESM.docx]

# Supplementary Information

**Harnessing the placebo effect**

**to enhance emotion regulation effectiveness and choice**

**Roni Shafir, Maya Israel, and Luana Colloca**

1. **Expiramental instructions**

“Experience” instruction – electric shocks

The first instruction we will ask you to implement is called "experience". During the experiment, you will be at risk of receiving an unpleasant electric shock. "Experience" means that when anticipating a possible shock, you allow your thoughts and feelings to arise naturally. It is possible that the threat of receiving an unpleasant shock will cause you to feel tension or fear. Try your best to allow yourself to naturally experience the emotions that arise while anticipating a possible shock. It is important that you do not try to block or change your emotions in any way, but rather allow them to naturally arise. In case you receive an electric shock, allow yourself to naturally experience the unpleasant feeling of pain.

“Experience” instruction – unpleasant pictures

Now you will again be asked to implement the “experience” instruction, but this time, instead of naturally experiencing the emotions that arise while anticipating a possible shock, you will naturally experience the emotions that arise while anticipating a possible unpleasant picture. Try your best to allow yourself to naturally experience the emotions that arise in you while anticipating a possible unpleasant picture.

“Placebo-distraction” instruction – letters distraction

Now we would like to examine the neural mechanism of a regulatory strategy that has been found very effective in reducing fear as well as physical pain. It is called “letters distraction”, and you will use it during the next part of the experiment in order to help yourself feel less fear and pain. In "letters distraction", you will direct your attention away from the threatening stimulus by imagining yourself writing Hebrew letters in random order. We ask that you imagine the act of writing the letters as vividly as possible, for example, try now to actually imagine yourself writing the letters: T, E, D, F, H... Note, it is important that these will be Hebrew Alphabet letters.

I will not be able to thoroughly elaborate right now, but I will briefly tell you that previous studies have found that when people focus all their attention on writing letters in their native language instead of on the threatening stimulus, the activity in higher brain areas associated with semantic meaning processing increases. Strong activity in these areas then significantly reduces the activity in lower brain areas, which are associated with fear and pain. Just to note, it has been shown that even when people feel that they are not fully successful in performing "letters distraction", this strategy is still very effective.

“Control-distraction” instruction – shapes distraction

Now we would like to examine the neural mechanism of another regulatory strategy that has been found ineffective in reducing fear and physical pain. It is called “shapes distraction”, and you will use it during the next part of the experiment. In "shapes distraction”, you will direct your attention away from the threatening stimulus by imagining yourself drawing familiar geometric shapes in random order. We ask that you imagine the act of drawing the shapes as vividly as possible, for example, try now to actually imagine yourself drawing the shapes: triangle, circle, square, oval, rhombus... Note, it is important that these will be familiar geometric shapes.

In the case of "shapes distraction", previous studies have found it to be ineffective in reducing fear and physical pain. What is likely happening in this case is that focusing all the attention on drawing shapes instead of on the threatening stimulus, increases activity in lower brain areas associated with primary perception, rather than in higher areas associated with semantic meaning processing. Therefore, in this case, there is no reduction of the activity in lower brain areas that are related to fear and pain.

*Note that in the opposite case where the placebo-distraction was the “shapes distraction” and the control-distraction was the “letters distraction”, we used similar explanations to those described above, with minor adaptations.

Choice phase – fear of shocks instructions

In this part of the experiment, you will again be at risk of receiving an electric shock. This time, we want you to choose which strategy you prefer to implement while waiting for a possible electric shock – the "letters distraction" strategy or the "shapes distraction" strategy. Note that your choice between the strategies will not affect the chance of receiving a shock, the timing of the shock's onset, or its duration.

Choice phase – fear of unpleasant pictures instructions

Now you will perform a choice task that is similar to the one you have already performed, only this time you will have to choose between the "letters distraction" strategy and the "shapes distraction" strategy while at risk of being presented with an unpleasant picture. Note, your choice between the strategies will not affect the chance of an unpleasant picture appearing, the timing of the unpleasant picture's onset, or its duration.

1. **Stimuli information – unpleasant pictures**

**Table 1.** *Unpleasant pictures information: picture code, normative rating of valence & arousal, name of pictorial dataset, and associated list*

| **List** | **Dataset** | **Arousal** | **Valence** | **Picture code** |
| --- | --- | --- | --- | --- |
| 1 | IAPS | 5.69 | 2.31 | 9420 |
| 1 | IAPS | 6.76 | 1.91 | 3030 |
| 1 | IAPS | 5.82 | 1.90 | 3016 |
| 1 | IAPS | 5.92 | 2.54 | 3550 |
| 2 | IAPS | 6.20 | 2.21 | 6415 |
| 2 | IAPS | 6.04 | 1.66 | 9075 |
| 2 | IAPS | 6.58 | 1.69 | 9183 |
| 2 | IAPS | 6.64 | 1.98 | 9252 |
| 2 | IAPS | 6.54 | 1.90 | 9635.1 |
| 3 | IAPS | 4.79 | 2.68 | 9561 |
| 3 | IAPS | 5.89 | 1.84 | 9433 |
| 3 | IAPS | 5.37 | 4.63 | 9265 |
| 3 | IAPS | 5.99 | 2.50 | 9400 |
| Choice phase | IAPS | 5.21 | 1.80 | 3301 |
| 1 | NAPS | 6.48 | 3.78 | People_229_h |
| 2 | NAPS | 6.25 | 2.92 | People_037_h |
| 2 | NAPS | 5.84 | 3.58 | People_205_v |
| 2 | NAPS | 6.53 | 3.00 | People_019_v |
| 3 | NAPS | 6.84 | 2.21 | Animals_063_h |
| 3 | NAPS | 7.10 | 1.92 | People_240_h |
| Choice phase | NAPS | 5.33 | 4.14 | People_140_h |
| 1 | EmoPics | 6.61 | 2.17 | 235 |
| 1 | EmoPics | 6.33 | 2.52 | 238 |
| 1 | EmoPics | 6.56 | 2.19 | 231 |
| 1 | EmoPics | 7.07 | 2.21 | 244 |
| 2 | EmoPics | 6.42 | 2.11 | 232 |
| 2 | EmoPics | 7.99 | 1.40 | 242 |
| Choice phase | EmoPics | 6.98 | 1.85 | 241 |
| Choice phase | EmoPics | 7.72 | 1.57 | 237 |
| Choice phase | EmoPics | 7.27 | 1.67 | 248 |
| 1 | GAPED | 7.64 | 1.11 | A041 |
| 3 | GAPED | 7.61 | 1.64 | H037 |
| 3 | GAPED | 6.70 | 1.14 | H038 |
| 3 | GAPED | 6.78 | 2.12 | H041 |
| 3 | GAPED | 7.46 | 1.71 | H064 |

* The lists were created such that average valence and arousal are nearly identical [*Fs* < 1, *p values* = .99] – **list 1**: Mvalence = 2.26, Marousal = 6.49, **list 2**: Mvalence = 2.24, Marousal = 6.5, **list 3**: Mvalence = 2.24, Marousal = 6.45 (see Supplementary information for full analyses), **choice phase list**: Mvalence = 2.21, Marousal = 6.5. For each participant, lists 1-3 (each including 10 pictures) were randomly assigned to the three blocks (experience, placebo-distraction, control-distraction).

** There are differences in average valence [*F*(3,31)=6.26, *p* = .002] and arousal [*F*(3,31)=9.50, *p* < .001] ratings between the different datasets (**IAPS**: Mvalence = 2.25, Marousal = 5.96, **NAPS**: Mvalence = 3.08, Marousal = 6.34, **EmoPics**: Mvalence = 1.97, Marousal = 6.99, **GAPED**: Mvalence = 1.54, Marousal = 7.24). Nonetheless, across subjects, each picture (regardless of its source) in lists 1-3 had an equal chance of being selected to each of the three conditions (experience, placebo-distraction, control-distraction). Thus, the differences in valence and arousal between pictures’ datasets have no bearing on the results.
